# Systematic Review of Plant Ribosome Heterogeneity and Specialization

**DOI:** 10.3389/fpls.2020.00948

**Published:** 2020-06-25

**Authors:** Federico Martinez-Seidel, Olga Beine-Golovchuk, Yin-Chen Hsieh, Joachim Kopka

**Affiliations:** ^1^ Willmitzer Department, Max Planck-Institute of Molecular Plant Physiology, Potsdam, Germany; ^2^ School of BioSciences, University of Melbourne, Parkville, VIC, Australia; ^3^ Biochemie-Zentrum, Universitaet Heidelberg (BZH), Heidelberg, Germany; ^4^ Bioinformatics Subdivision, Wageningen University, Wageningen, Netherlands

**Keywords:** functional heterogeneity, substoichiometry, specialized ribosome, ribosomal code, remodeling

## Abstract

Plants dedicate a high amount of energy and resources to the production of ribosomes. Historically, these multi-protein ribosome complexes have been considered static protein synthesis machines that are not subject to extensive regulation but only read mRNA and produce polypeptides accordingly. New and increasing evidence across various model organisms demonstrated the heterogeneous nature of ribosomes. This heterogeneity can constitute specialized ribosomes that regulate mRNA translation and control protein synthesis. A prominent example of ribosome heterogeneity is seen in the model plant, *Arabidopsis thaliana*, which, due to genome duplications, has multiple paralogs of each ribosomal protein (RP) gene. We support the notion of plant evolution directing high RP paralog divergence toward functional heterogeneity, underpinned in part by a vast resource of ribosome mutants that suggest specialization extends beyond the pleiotropic effects of single structural RPs or RP paralogs. Thus, Arabidopsis is a highly suitable model to study this phenomenon. Arabidopsis enables reverse genetics approaches that could provide evidence of ribosome specialization. In this review, we critically assess evidence of plant ribosome specialization and highlight steps along ribosome biogenesis in which heterogeneity may arise, filling the knowledge gaps in plant science by providing advanced insights from the human or yeast fields. We propose a data analysis pipeline that infers the heterogeneity of ribosome complexes and deviations from canonical structural compositions linked to stress events. This analysis pipeline can be extrapolated and enhanced by combination with other high-throughput methodologies, such as proteomics. Technologies, such as kinetic mass spectrometry and ribosome profiling, will be necessary to resolve the temporal and spatial aspects of translational regulation while the functional features of ribosomal subpopulations will become clear with the combination of reverse genetics and systems biology approaches.

## Introduction

Historically, ribosomes have been considered passive mediators of the central dogma of molecular biology. Nonetheless, the first concept of the role of ribosomes in molecular information flow proposed in 1958 (Crick, 1958; Crick, 1970), was based on the “one gene one ribosome one protein” hypothesis. This notion implied an extreme degree of ribosome specialization. Later, in 1961, the discovery of mRNA as a carrier of open reading frames (ORFs) that code for protein synthesis marginalized the ribosome as a passive bystander of translation (Brenner et al., 1961). The role of ribosomes started to be reconsidered between 1985 and 1995, when independent studies supported the view that the heterogeneity of ribosome composition is likely an additional layer of translational regulation. In 1987, two divergent 18S rRNA sequences were found to be dominant during distinct stages of the rodent malaria life cycle (Gunderson et al., 1987). In 1990, ribosomal protein (RP) expression and posttranslational modification (PTM) were found to change in *Dictyostelium discoideum* upon transition from a unicellular to a multicellular lifestyle (Ramagopal, 1990). In 1995, the model plant *Arabidopsis thaliana* revealed tissue-specific expression of the many RP paralogs that exist in plants (Williams and Sussex, 1995). Nowadays, among many examples, well-studied global translational regulators in plants couple external stimuli to translation, arguing for deeper investigation of translational control (Urquidi Camacho et al., 2020) upon environmental cues.

The altered composition of the translation machinery at any level is a phenomenon called ribosome heterogeneity (Horiguchi et al., 2012; Shi et al., 2017; Gerst, 2018). Ribosome heterogeneity includes sequence variation of rRNAs, absence of specific RPs from the canonical ribosome structure, which causes substoichiometric ribosomes, exchange of RP paralogs, posttranscriptional or posttranslational modifications of rRNA or RPs and possibly additional variations of the ribosome-associated proteome. The difference between heterogeneity and specialization resides in the functional role assigned to sub-ribosomal populations. Thus, specialized ribosomes are defined as a subset of heterogeneous ribosomes that constrain translation to specific mRNAs or may have other specific functions. Functional subpopulations of ribosomes would appear for example after an environmental cue to shape the acclimated proteome. These definitions have previously been proposed (Emmott et al., 2018; Genuth and Barna, 2018) and reflect controversial opinions in the field as yet.

Currently, there is a dualism of hypotheses. The first hypothesis states that heterogeneous ribosomes translate mRNA subsets using mechanisms linked to the diverse aspects of structural ribosome heterogeneity. The second suggests that preference of translation toward transcript subsets is a consequence of insufficient amounts of functional ribosomes. The insufficiency hypothesis considers ribosomes as static machines and assigns selective properties of preferred translation to transcripts. Highly translated mRNAs are thought to out-compete less readily translated but required transcripts when availability of functional ribosomes limits translation. A similar dualism of hypotheses prevails among explanations of phenotypes linked to *rp*-paralogs where the term of ribosome insufficiency was coined for plants (Horiguchi et al., 2012). In this context, paralog mutant abnormalities are attributed to insufficient functional ribosomes and not to specialized functions of heterogeneous ribosomes. The lack of information on the highly resolved spatiotemporal ribosome composition and the ribo-interactome limits our ability to distinguish between these alternative hypotheses. To fully understand what constitutes functional ribosome heterogeneity, technical obstacles must be surpassed.

RP substoichiometry is likely to assist specialization (Slavov et al., 2015). In yeast, the central role of RPs during translational regulation supports the existence of a ribosomal code (Komili et al., 2007), i.e., the concept of an additional level of complexity attributed to ribosomes that regulate protein translation, and is paralleled by the concept of a histone code that contributes to the regulation of the transcriptional status of a gene. Specialization may entail the remodeling of existing ribosomes where the core structure of the ribosome will be reused and the surface and solvent-exposed proteins are exchanged by *de novo* synthesized paralogs. Alternatively, new ribosomal populations may be *de novo* synthesized. These processes may give rise to substoichiometric ribosome populations in the cell. In plants, where each RP family contains several paralogs, we suggest extending and generalizing the term substoichiometric ribosome population to include ribosomes with exchanged RP paralogs. Currently, analytical methods capable of monitoring specialization are scarce. Therefore, claims of new findings in the field are technology dependent and must be interpreted carefully.

In this review, we distinguish and discuss ribosome heterogeneity according to structural components starting with interacting factors during ribosome biogenesis. Ribosome synthesis represents a compendium of steps by which specialized ribosomes may become assembled. Additionally, we review the methods used for generating insights into ribosome specialization. Our biological focus is on the adaptive benefit of potential functional heterogeneity of cytosolic ribosomes modulating stress responses of sessile organisms, such as plants. Our technical focus defines suitable methodological strategies that will approximate or even allow the acceptance or rejection of ribosome specialization. In all these aspects, we use plants as potentially important but neglected models of ribosome function.

## Assembly of Heterogeneous Ribosomes

Cytosolic ribosomes in eukaryotes consist of a 60S large subunit (LSU) and a 40S small subunit (SSU). The latter decodes mRNA, and the former catalyzes the peptidyl transferase reaction that leads to the peptide bond formation of the newly synthesized proteins. The subunits are composed of rRNA and accessory ribosomal proteins (RPs). The large subunit is composed of 5S, 5.8S, and 25S rRNA, which ranges between 25S and 26S in plants but is 28S in mammals (Chang et al., 2005). In contrast, the small subunit contains only a single 18S rRNA. At the protein level, the plant 60S and 40S subunits contain at least 47 and 33 RPs, respectively (Wilson and Cate, 2012), with each RP encoded by two to seven paralogs (Barakat et al., 2001; Browning and Bailey-Serres, 2015) **(** **Supplemental Table S1** **).** Thus, the 80 RP families may comprise 10^34^ different potential ribosome structural conformations that, considering paralog number, could theoretically serve as a source of heterogeneity (Hummel et al., 2012) and may be the basis of functional specialization or functional divergence within RP families. Given this, specialized ribosomes seem more likely than ribosome heterogeneity seen as a purely stochastic non-functional phenomenon. Important unresolved questions need to be answered: If heterogeneity is basis of a functional mechanism in plant cells, how is it controlled and when is it triggered? A first indication of heterogeneity as a non-stochastic process may be considered from the observation that core ribosomal proteins are assembled by a controlled and highly sequential biogenesis process. Hence, if the assembly line is better understood, then we could improve our current knowledge of ribosome specialization.

**Figure 1:**
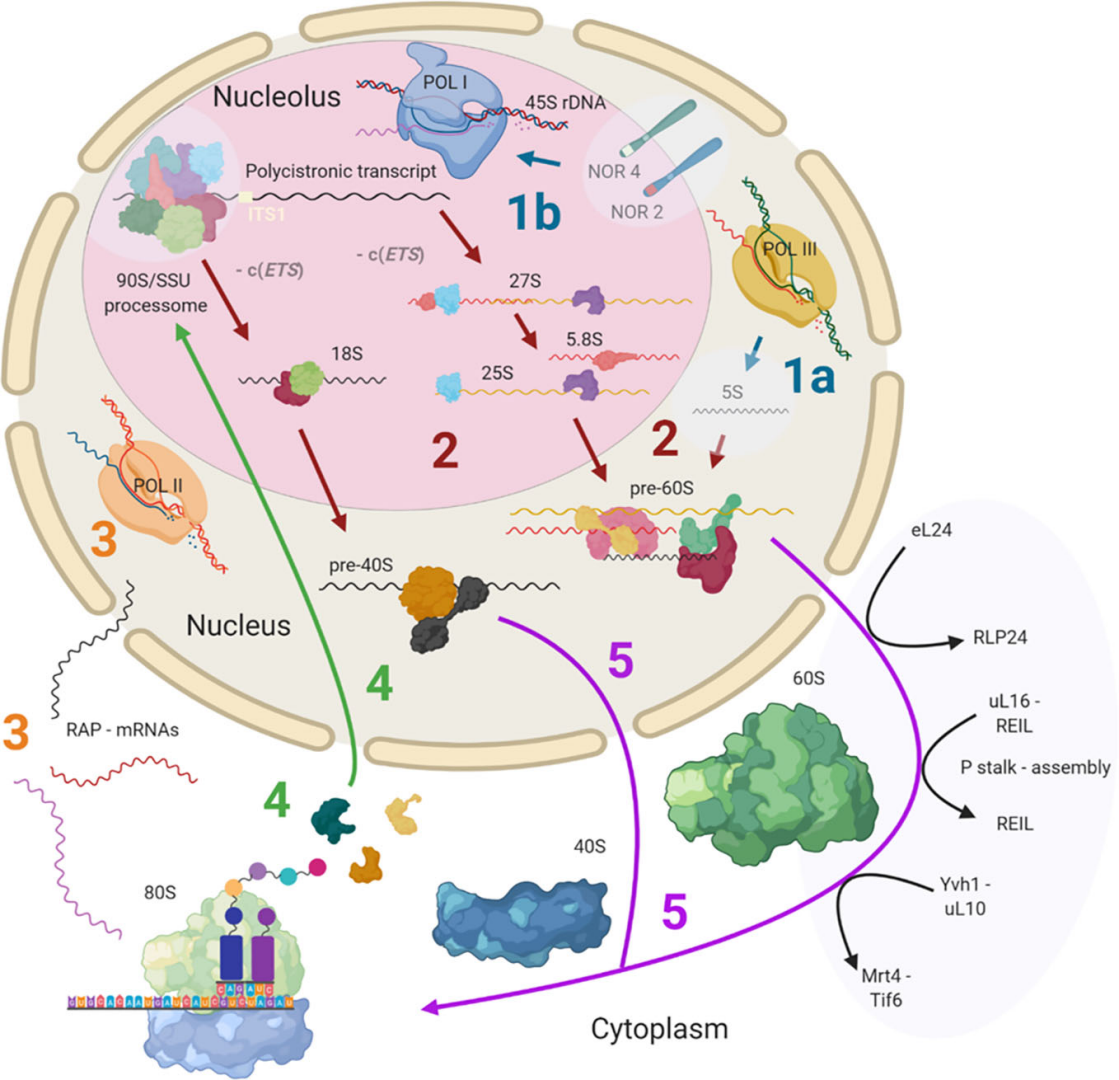
Simplified scheme of plant cytosolic ribosome biogenesis highlighting the potential steps at which structural heterogeneity may occur and can be controlled. Biogenesis is complex and involves at least three cell compartments, the nucleolus, the nucleoplasm, and the cytoplasm. (Step 1A) 5S rRNA is transcribed by RNA polymerase III (POL III) in the nucleus. (Step 1B) The 45S rDNA, localized in the nucleolus, is transcribed into a polycistronic transcript containing 18S, 5.8S and 25S rRNAs by RNA polymerase I (POL I). Heterogeneity may rise from rDNA loci coding for different rRNA species. (Step 2) The large pre-rRNA transcript forms the 90S pre-ribosome/SSU processome, a large ribonucleoprotein complex, which is processed into pre-40S and pre-60S subunits after the splicing event on ITS1. Biogenesis factors are temporarily recruited and ribosomal proteins (RPs) are permanently assembled while rRNA is successively processed. Heterogeneity may result from the recruitment of different rRNAs and ribosome-associated proteins (RAPs), including RPs and RP paralogs. (Step 3) The RAPs are transcribed by RNA polymerase II (POL II). Heterogeneity can result from the changed availability of transcripts for subsequent translation or the presence of different splicing variants. (Step 4) RPs and other RAPs are translated in the cytoplasm and imported into the nucleus where they are assembled or assist the assembly process. Heterogeneity may be caused by availability of divergent RAP and RP paralogs at the time and location of assembly within the nucleus. (Step 5) The nuclear ribosomal pre-subunits are exported to the cytoplasm where they undergo the final maturation steps that render the subunits translationally competent (black arrows). Heterogeneity may arise from the last steps of ribosome biogenesis mediated by RAPs. Posttranscriptional or posttranslational modifications of all components may occur at any stage during or post ribosome biogenesis. Note that some processes and or structure-derived insights have yet to be described in plants (light blue transparencies highlights), and these gaps have been filled with knowledge from other model eukaryotes. The figure was created with BioRender (www.biorender.com) and exported under a paid subscription. 5’ or 3’ external transcribed spacer c(ETS), internal transcribed spacer (ITS), nucleolar organizer regions (NORs).

Ribosome biogenesis has previously been reviewed both for plants (Weis et al., 2015a; Sáez-Vásquez and Delseny, 2019) and yeast (Woolford and Baserga, 2013). This review provides detailed insights into known and currently unknown plant ribosome biogenesis aspects and is focused on highlighting the processing steps and structures, which may contribute to the assembly of heterogeneous ribosomal populations. Cytosolic ribosome biogenesis starts in the nucleolus and finalizes in the cytoplasm where the last maturation steps take place (**Figure 1**). The main steps at which ribosome heterogeneity may be introduced and specialized functions may be controlled are: 1) 45S and 5S rDNA transcription, 2) pre-ribosomal RNA (pre-rRNA) processing, 3) transcription of RPs and ribosome-associated proteins (RAPs) such as plant ribosome biogenesis factors (RBFs) (Weis et al., 2015a; Palm et al., 2019) or translation factors (Browning and Bailey-Serres, 2015), 4) RP and RAP translation and reallocation to the nucleus, and finally, 5) successive RP and RAP assembly during ribosomal subunit maturation. These key processing steps throughout biogenesis may serve as points of control for the generation of specific ribosome populations (**Figure 1**).

### Variation of Ribosomal RNA

Cytosolic ribosomes comprise four mature rRNAs, i.e., 5S, 5.8S, 25S, and 18S. The process to obtain the mature RNAs starts with:

RNA polymerase III mediated synthesis of 5S rRNA (**Figure 1** Step 1A). In Arabidopsis, 5S genes are encoded by over 2000 copies distributed over three locations on chromosomes 3, 4, and 5 (Murata et al., 1997). Loci in different chromosomes encode rRNAs of varying lengths (Murata et al., 1997; Poczai et al., 2014), and are differentially enriched by epigenetic marks promoting specific chromatin states. The balance between euchromatin and heterochromatin impacts which 5S rRNAs get transcribed (Vaillant et al., 2007). A locus on chromosome 5 gives rise to an atypically long 5S splicing variant due to aberrant transcription termination, which is also expressed in several mutants deficient in chromatin remodeling processes (Vaillant et al., 2006; Blevins et al., 2009). 5S rRNA genes from this locus translocated in Arabidopsis ecotype Ler, impacting chromatin status and ultimately the selected 5S loci that get transcribed (Simon et al., 2018). Similarly, if 5S rDNA chromatin gets remodeled following stress cues (Asensi-Fabado et al., 2017), a transition could be initiated to modulate ribosome subpopulations. Moreover, the translocation events have increased the exchange frequency among 5S rDNA loci (Simon et al., 2018), increasing the possibilities of coupling the right locus with the right environmental stimulus, ultimately converging at a functionally advantageous ribosome.

In parallel, RNA polymerase I mediated synthesis of a polycistronic rRNA transcript, the precursor of 18S, 5.8S and 25S rRNA (**Figure 1** Step 1B), from highly duplicated 45S rDNA genomic repeats (Sáez-Vásquez and Echeverría, 2007). The tandem-repeated units are arranged into nucleolar organizer regions (NORs) on the short arms of chromosomes 2 and 4 of *Arabidopsis thaliana* (Copenhaver and Pikaard, 1996; Poczai et al., 2014; Browning and Bailey-Serres, 2015). Both NORs contain 45S rDNA variants, with those on chromosome two being tightly regulated during plant development (Chen and Pikaard, 1997; Fransz et al., 2002; Mohannath et al., 2016; Sáez-Vásquez and Delseny, 2019). The short arms of human 21 and 22 NOR-containing chromosomes are physically embedded in the nucleolus (Dunham, 2005).

Remodeling of pre-existing ribosomes by exchanging rRNA seems unlikely, since this process would require fundamental ribosome disassembly and reassembly. Hence, specialized rRNAs may be introduced by *de novo* ribosome synthesis upon an environmental challenge. For example, a controlled mechanism of *Vibrio vulnificus* Gram-negative bacteria upon temperature or nutrient shifts, synthesizes divergent rRNAs that ultimately direct translation of specific mRNAs (Song et al., 2019). Similarly, variation of the rRNA nucleotide sequence modulates the stress responses of *Escherichia coli* in the newly synthesized active translating fractions of ribosomes (Kurylo et al., 2018). An alternative non-plant example from *Escherichia coli* and human studies, are posttranscriptional modifications of rRNA (Popova and Williamson, 2014; Natchiar et al., 2017), which are concomitant to RP substoichiometry (Popova and Williamson, 2014). These modifications can confer selectivity to ribosomes. In plants, these mechanisms remain to be found. However, examples of rRNA heterogeneity harboring functional potential exist both at rDNA level, as outlined in the two previous paragraphs, and during rRNA pre-processing, as detailed in the following section.

### Alternative Pre-Ribosome Processing

The 5S rRNA transcript is processed in the nucleoplasm (**Figure 1** Step 2). In contrast, the initial steps of polycistronic rRNA processing take place in the nucleolus. After 45S rDNA transcription, the resulting transcript, designated as 35S pre-rRNA in yeast, associates with a larger ribonucleoprotein complex forming the 90S pre-ribosome and commences processing steps in the nucleolus (**Figure 1** Step 2). The 90S pre-ribosome complex contains similar components as the SSU processome (Grandi et al., 2002). This initial ribosome maturation complex was purified from other eukaryotes and provided structural insights into the initial pre-rRNA processing steps (Kressler et al., 2017). While it remains to be structurally characterized in plants, most protein orthologs within the complex are encoded in the plant genome (Sáez-Vásquez and Delseny, 2019). The 90S/SSU-processome, also designated as U3 snoRNP (Sáez-Vasquez et al., 2004), likely exchanges RBFs and assembles RPs, since 22 homologues of *Arabidopsis thaliana* SSU RPs co-purified with the BoU3 complex of *Brassica oleracea* as was determined by mass spectrometry of 1D SDS-PAGE purified bands (Samaha et al., 2010). Hence, specialized RPs or paralogs may already be assembled at an early stage of ribosome biogenesis. In plants, the complex is thought to cleave the internal transcribed spacer (ITS), ITS1, facilitated by previous trimming and cleavage at the P site (Zakrzewska-Placzek et al., 2010), that is, the primary endonucleolytic cleavage site located in the 5’ external transcribed spacer (ETS). ITS1 cleavage splices the polycistronic transcript into 27S rRNA, containing the immature 25S and 5.8S rRNAs, and 18S rRNA, thereby splitting the processing into a pre-60S and pre-40S branch. Most unprocessed Arabidopsis 35S-type transcripts contain a non-conserved insertion of 1,083-bp that is absent from other cruciferous species (Sáez-Vasquez et al., 2004). This feature supports the notion of unique features of Arabidopsis ribosome biogenesis as compared to other plant species. Such unique aspects should be taken into account when interpreting and generalizing studies of 35S-type transcript pre-processing in Arabidopsis.

Pre-rRNA processing of the polycistronic transcript follows two independent pathways in plants (Weis et al., 2015b). This process has been regarded as a redundancy that may secure ribosome abundance under varying conditions. The convergence point of the alternative processing paths is the 27SB_S/L_ rRNA, which is the 27S pre-rRNA spliced from any 5’ or 3’ ETS or -c(ETS) as depicted in **Figure 1**. At this convergence point, the pre-60S subunits are released into the nucleoplasm (Gadal et al., 2002) for further processing. The small subunit 18S rRNA processing may converge at a barely detectable 20S pre-rRNA that is produced after the excision of 5.8S by MTR4 (Lange et al., 2011) and a similar ETS splicing as aforementioned for pre-60S. rRNA processing involves plant-specific RBFs (Palm et al., 2019), suggesting that specialized features of ribosome biogenesis are to be found in plants. This supports the view that RBFs and RPs have specialized functions in alternative pre-rRNA processing routes in plants. Examples indicate that mutations in Arabidopsis AtBRX1-1 and AtBRX1-2 orthologs of the yeast RBF, Brx-1, affect only one of the alternative pre-rRNA processing routes (Weis et al., 2015b). In yeast, Brx-1 associates with RBFs Tif6 and Ebp2 to form the Rpf2 complex (Talkish et al., 2012), which also contains structural proteins uL18 (ScRPL5) and eL18 (ScRPL11). In Arabidopsis, *tif6* and *brx1-1* transcripts are differentially accumulated compared to wild type (WT) in mutant lines of the RBF REIL that are likely impaired in late cytosolic ribosome maturation and during cold acclimation (Beine-Golovchuk et al., 2018). Similarly, heat stress could decrease the abundance of pre-rRNAs belonging to one of the alternative processing pathways (Weis et al., 2015a). More generally, plant responses to abiotic stress include altered expression patterns of pre-rRNA processing factors. Such expression changes occur mainly during cold, heat and UV-B light stresses (Sáez-Vásquez and Delseny, 2019). In summary, beyond securing ribosome abundance by redundant factors, evidence points toward effective subfunctionalization and specialized mechanisms that act during stress and enable pre-rRNA processing.

Following nucleolar and nuclear processing, pre-60S LSU and pre-40S SSU complexes are exported into the cytoplasm. Pre-LSU is aided by RBFs to undergo final maturation steps (**Figure 1** Step 5). The associated factors have been elucidated and reviewed in yeast (Woolford and Baserga, 2013; Greber et al., 2016; Ma et al., 2017). The cytosolic steps in plants are thought to be mediated by cytosolic RBF homologs, amongst them REIL1 and REIL2 (Beine-Golovchuk et al., 2018). REIL proteins are Arabidopsis RBFs homologous of yeast Rei1. In yeast, Rei1 has a structural proofreading function of the 60S LSU subunit (Meyer et al., 2010; Greber et al., 2016). During cytosolic LSU maturation in yeast, a RLP24 placeholder protein is replaced by RP eL24, then RP uL16 is added and P-stalk assembly is initiated in parallel to or after Rei1 action (Meyer et al., 2010). The P-Stalk is a pentameric uL10-(P1-P2)_2_ complex in yeast (Wawiórka et al., 2017), with additional P3 components in plants, that assists translation associated GTPases. For P-stalk assembly, Yvh1 mediates the release of Mrt4, a placeholder for uL10, and enables substitution by functional uL10 (Zhou et al., 2019). In rice blast fungus *Magnaporthe oryzae,* MoYvh1 is translocated to the nucleus upon oxidative stress where it interacts with MoMrt4 in a process that ultimately subverts the production of proteins needed for plant immunity (Liu et al., 2018), implying that these maturation factors could guide biogenesis of specialized ribosomes to filter immunity-related proteins. After final quality control checks, ScTif6, the anti-SSU-LSU association factor (Basu et al., 2001), is released, and the 60S subunit is rendered translationally competent.

### Variation of Ribosome Associated Proteins

During the whole biogenesis process, ribosome associated proteins or RAPs are either transiently (i.e., proteins assisting the process) or in the case of RPs, permanently (i.e., proteins comprising structural constituents of translationally competent complexes) bound to the pre-ribosomes. The RP and other RAP coding genes are transcribed and spliced in the nucleus (**Figure 1** Step 3), the mRNAs are exported and translated in the cytoplasm and finally, most of the RAPs are imported into the nucleus and nucleolus for ribosome assembly (**Figure 1** Step 4).

**Figure 2:**
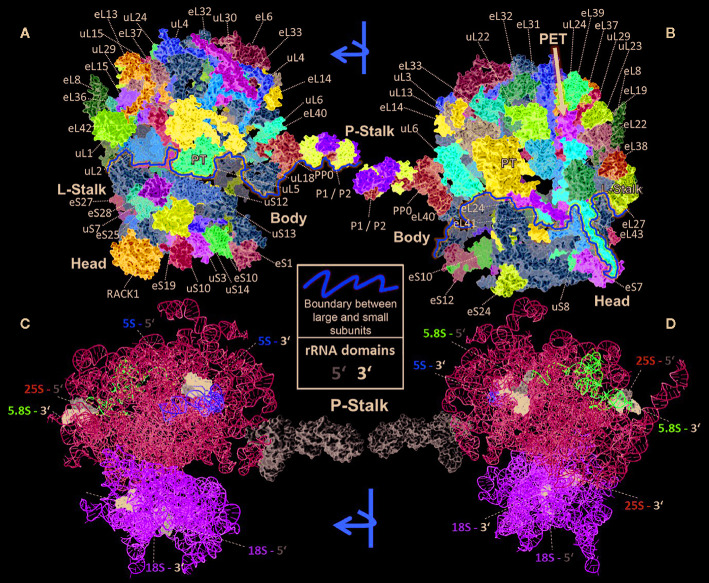
Localization of RPs (top) or rRNAs (bottom) within the translating wheat 80S ribosome (Armache et al., 2010a; Armache et al., 2010b; Ben-Shem et al., 2011; Gamalinda et al., 2013) visualized by PyMOL (PyMOL, RRID : SCR_000305). RP localization was modeled at 5.5 Å resolution by combining structural data of wheat and yeast according to (Ben-Shem et al., 2010). rRNA chains are omitted from top panels **(A, B)** and are shown separately in bottom panels **(C, D)** without RP decorations except the P-Stalk that was added as a white surface outline for orientation. Each colored amino acid chain represents the position of a ribosomal protein family within the 40S SSU (lower part of the 80S ribosome) or in the 60S LSU (upper part). **(A)** Ribosome solvent surface. The main topological characteristics, P-stalk, L-stalk, head and body, and the 40S SSU to 60S LSU interface are indicated (yellow line). **(B)** Rotated ribosome. The interface and location of the polypeptide exit tunnel (PET, arrow) are indicated. **(C, D)** Rotated ribosome solvent and interface positions featuring the rRNA chains distinguished by colors. Positions of the four 5’ (grey) and four 3’ (black) ends of the 18S, 25S, 5.8S and 5S rRNAs are indicated by highlighting of the last ten nucleotide residues.

All RPs have specific entry points during ribosome biogenesis. Therefore, the main processing steps of ribosome biogenesis may determine when RP-specialized ribosomes can be assembled based on selection of specific RPs or paralogs instead of a non-controlled stochastic choice. Controlled assembly would mean that adjacently located RPs, if co-assembled, might be co-dependent on each other or on specific biogenesis factors. Consequently, defined ribosomal regions might be modulated by specialization mechanisms that rely on a sequential assembly line to construct functionally divergent complexes. In line with the previous idea, systematic analyses of individual ribosomal protein mutants (*rp*), compiled in a literature review of yeast ribosome biogenesis (Woolford and Baserga, 2013), have shown a correlation between localization of RPs (**Figures 2A, B**) relative to rRNA domains (**Figures 2C, D**) and the impairment of pre-rRNA maturation. For example, SSU proteins can be attached near the 5´ or 3´ domains of 18S rRNA, which are located at the body and head of the SSU, respectively. RPs near the 5´ end are important during the early stages of pre-rRNA processing, while those near the 3´ end are incorporated in later maturation steps. Similarly, LSU RPs are docked to three rRNA regions. 1) Domains I and II, approximately surrounding the equator of the solvent-exposed face of the LSU, are near the 5´ ends of 25S rRNA and 5.8S rRNA, respectively. The RPs binding near these ribosomal regions are necessary for 27SA_2_ and 27SA_3_ pre-rRNA processing. 2) Domains I and III are located near the polypeptide exit tunnel and the RPs binding nearby are necessary for 27SB pre-rRNA cleavage. 3) Finally, the third docking area is located near the central protuberance on the interface side of the LSU. The nearby bound RPs are necessary for 7S pre-rRNA processing and nuclear export. Whether plant-RPs conserve these sequential and spatial dependencies, remains to be tested.

Transiently bound RAPs assist the translational machinery at every step, from ribosomes biogenesis, through translation, to ribosome recycling. All of these RAPs comprise the ribo-interactome that is highly complex and includes multiple RAP paralogs. Presence of RAP paralogs in plants implies that sub- or neofunctionalized RAPs may mediate cell responses by selective translation as was demonstrated for the mammalian ribo-interactome (Simsek et al., 2017). RAPs, such as the subset of RBFs, can have a wide range of functions during assembly and processing. For example, the mammalian ribo-interactome contains RAPs such as mRNA binding proteins, mRNA/tRNA modifiers, RNA helicases, and potential regulators of metabolism and the cell cycle (Simsek et al., 2017). In plants, numerous examples indicate specific or specialized RAPs. The presence of plant-specific factors, such as the additional eIFiso4F cap-binding complex (Browning et al., 1992; Browning and Bailey-Serres, 2015), which has functionally divergent isoforms (Gallie, 2016), may serve as the first indicator. For example, mutations of the rice eIFiso4F homolog confer resistance to yellow mottle virus (RYMV) (Albar et al., 2006), suggesting that functional divergence of this factor is readily adaptable to generate selective translation constrains. A second indication is the absence of 25% of yeast RBF orthologs from the plant genome (Weis et al., 2015a). The missing RBFs were likely replaced during plant evolution. A third indication is the duplication of conserved RBFs in plants, such as BRX1-1/1-2, Lsg-1/2, NUC1/2, XRN2/XRN3, and REIL1/2 (Weis et al., 2015a) that are, as was explained above, involved in multiple stress responses of pre-ribosome processing. Clearly, the ribo-interactome of plants is more complex than in other model eukaryotes. This complexity has already resulted in subfunctionalization as may be exemplified by the two REIL biogenesis factor paralogs that act in the cytosol. Only REIL2 but not REIL1 is required for successful cold acclimation. Absence of both paralogs, however, enhances the defect and halts Arabidopsis development and growth at low temperature (Schmidt et al., 2013; Beine-Golovchuk et al., 2018). REIL proteins affect accumulation of non-translational ribosomal complexes (Cheong et al., 2020), that is, free pools of 60S LSUs and single 80S monosomes. and in the case of REIL2 are linked to the cold induced C-repeat-binding factor (CBF) regulon (Yu et al., 2020), which is a compendium of more than 100 genes with altered expressions due to the action of CBF transcription factors and enhances freezing tolerance. Whether REIL functions include contributions to ribosome specialization and cold-acclimated ribosome biogenesis are hypotheses that remain to be validated.

## Protein Composition of the Cytosolic Ribosome

An early attempt to characterize an eukaryotic, cytosolic ribosomal proteome (CRP) was conducted with rat liver ribosomes (Wool et al., 1995). The 79 RP families that were characterized had homologs in yeast and plants (Wilson and Cate, 2012). Each yeast protein is encoded by two paralogs. Only 64 of the yeast RPs are essential for growth (Steffen et al., 2012). There is an additional 80^th^ plant-specific RP family (Carroll, 2013), namely, the acidic stalk protein P3 (Barakat et al., 2001; Chang et al., 2005; Carroll, 2013). The other 79 plant families represent yeast homologs with high fidelity. Despite the high similarity between the eukaryotic CRPs, in plants, duplication led to structural divergence between RP paralogs (Wool et al., 1995; Barakat et al., 2001). Attempts to verify plant RP families that were predicted at the genome level through proteomic approaches have produced a range of answers. For example, a proteomic study found representatives of all 80 plant RP families, with specific identifications of 87 family members (Carroll et al., 2008). A more recently published data set mapped 70 RP families and 165 RP family members to the CRP (Hummel et al., 2015). The striking difference in the detected paralogs per RP family in both studies may be explained by technical variation of the complex CRP preparation and proteomic analysis but may equally likely originate from changes of ribosome heterogeneity between the two investigated ribosome populations. The most recent study that refined the compositions of the Arabidopsis cytosolic ribosome mapped 76 RP families and 184 members using a label-assisted proteomics approach (Salih et al., 2019).

### Deviation From Canonical Compositions

If substoichiometric complexes arise from a non-random specialized ribosome biogenesis, and RPs get affected as co-assembled groups, we need to be able to map the changes of RPs or RP paralogs onto an accurate plant ribosome structure in order to understand the spatial boundaries of these modulatory mechanisms. The currently best localization of RPs within a cytosolic plant ribosome was generated through modelling of known ribosomal protein structures (using archaeal and bacterial templates) into a bread wheat *Triticum aestivum* cryogenic electron microscopy (cryo-EM) map that was reported at 5.5 Å resolution (Armache et al., 2010b). Simultaneously, the rRNA backbone structures were elucidated at 5.5 Å resolution and comprehensively modeled (Armache et al., 2010a), thus completing the current structure model of the wheat 80S ribosome. We coupled RP localization with a comprehensive mapping of RP and RP paralogs (**Supplemental Table S1**) to ribosome complexes (**Figure 2**), compiled based on model organisms, such as yeast (Ben-Shem et al., 2011; Gamalinda et al., 2013). **Figure 2** summarizes the main structural domains and location of RPs by rotated 2D projections of the 3D wheat 80S translating ribosome model. The *Triticum aestivum* structure contains 80 ribosomal proteins, 47 of the 60S subunit and 33 of the 40S subunit, as well as 4 rRNA structures. Given that a high resolution structure of the mature, translating Arabidopsis cytosolic ribosome has yet to be made publically available, the *Triticum aestivum* 80S ribosomal structure published by Armache et al. (2011) was used as reference for our visualizations (PDB ID 4v7e). Using protein BLAST comparisons, we verified the RP identity of Arabidopsis RP homologs of the protein entries linked to the macromolecular Crystallographic Information Files (mmCIF) of the wheat 80S structure model. We concluded that Arabidopsis RPs (**Supplemental Table S1**) are adequately matched to the wheat RPs mapped in the 80S structure model. To the best of our knowledge, the wheat structure is the currently most complete and adequate, high-resolution plant cytosolic ribosome structure in the PDB database and represents the current canonical structure model of plant 80S ribosomes.

Several lines of evidence indicate that deviations from the canonical 80S structure of plant ribosomes exist, that is, incomplete, substoichiometric ribosomes lacking RPs or ribosomes with varied RP paralog composition (**Table 1**). RP-dependent ribosome structural divergence was deduced by shifts in mass or charge among 25% of the Arabidopsis RPs analyzed (Chang et al., 2005). These observations can be caused by paralog exchanges or by PTMs. Paralog exchanges are likely considering independent reports showing that paired transcript translation and protein degradation rates of cytosolic-RPs from tomato *Solanum lycopersicum* are high (Belouah et al., 2019) and cytosolic RPs of Arabidopsis have a high standard deviation of the protein degradation rates (Li et al., 2017a). These studies suggest that a potential mechanism of ribosome remodeling exists even though RPs are in general stable and long-lived (Li et al., 2017a). Ribosomal complexes of Arabidopsis have a mean-RP half-life of 3-4 days (Salih et al., 2019). Considering the general stability of ribosomes, it seems likely that the high variation among RPs results from induced translation targeted to specific RPs or RP paralogs and remodeling of pre-existing ribosome complexes by RP exchange.

**Table 1:** Plant studies with supporting evidence for and major conclusions regarding cytosolic ribosome heterogeneity and specialization in chronological order.

Study	Species	Evidence	Major Conclusions
(Chang et al., 2005)	*Arabidopsis thaliana*—Cell culture	Proteomics	25% of the cytosolic RPs vary in terms of mass or charge, affecting the overall composition of the 80S monosome.
(Degenhardt and Bonham-Smith, 2008a; Degenhardt and Bonham-Smith, 2008b)	*Arabidopsis thaliana*	Reverse genetics, live cell imaging, RNA interference, transcript profiling	RP paralog AtRPL23aA (uL23) is targeted to the nucleolus. Loss of the paralog causes pleiotropic effects associated with an *rp* plant mutant. AtRPL23aB is targeted to the nucleus but its absence does not cause developmental or growth abnormalities. Dosage compensation does not apply to paralog loss in uL23.
(Guo and Chen, 2008)	*Arabidopsis thaliana*	Reverse genetics, mutant complementation	AtRACK1B and AtRACK1C loss of function mutants do not have the growth and developmental abnormalities that AtRACK1A has. Multiple AtRACK1 mutants exacerbate the abnormalities. The B and C paralogs complement loss of function of paralog A.
(Whittle and Krochko, 2009)	*Brassica napus*—Microspores, ovules, pollen, microspore-derived embryos, and *in vitro* pollen	Transcriptome co-expression networks	*Brassica napus* has a tissue-specific RP paralog composition, which is likely associated with tissue differentiation and/or specialization.
(Falcone Ferreyra et al., 2010)	*Arabidopsis thaliana*—Shoots	Label-free proteomics	UV-B stress differentially regulates paralogs from the uL16 family of Arabidopsis. RPL10C is upregulated by UV-B in all studied organs, while AtRPL10B is downregulated and RPL10A does not change upon a UV-B stimulus.
(Szick-Miranda et al., 2010)	*Arabidopsis thaliana*	Reverse genetics, RT-qPCR, phenotyping	Type II uS8 RP paralogs are plant specific and evolutionarily divergent. RPS15aB and RPS15aE are differentially expressed. RPS15aE mutant has larger leaves, roots, and cells.
(Rosado et al., 2010)	*Arabidopsis thaliana*	Reporter gene microscopy	Subpopulations of RPL4-containing heterogeneous ribosomes co-exist featuring paralog A or D.
(Sormani et al., 2011b)	*Arabidopsis thaliana*	Transcriptomic data	Subgroups of RPs corresponding to specific paralogs are transcriptionally regulated during stress, leading to “ribosome diversity”. The authors propose a model that controls heterogeneity during biogenesis.
(Hummel et al., 2012)	*Arabidopsis thaliana*—Shoots	Transcriptomic data and label-free proteomics	Sucrose feeding induces abundance changes in specific paralogs, among them eL28 (AtRPL28A) and eS7 (AtRPS7C). Additionally, at transcript level, many RP genes become upregulated.
(Falcone Ferreyra et al., 2013)	*Arabidopsis thaliana*—Shoots	Proteomics, subcellular localization, yeast complementation	Non-redundant functional roles of uL16 RPs are indicated. RPL10C expression is restricted to pollen grains. RPL10B localization to the nuclei increases after UV-B stress. The three isoforms complement a yeast *uL16* mutant.
(Wang et al., 2013)	*Arabidopsis thaliana*—Roots	Transcriptomic & label free proteomics	Specialized paralogs are associated with Pi-deficiency, uL11 (AtRPL12B) regulated at protein level, eL33, eL39, uS9 (AtRPL35aC, AtRPL39B and AtRPS16B) regulated at transcript level, or with Fe-deficiency, eL22 (AtRPL22B and AtRPL22C) regulated at protein level.
(Gamm et al., 2014)	*Arabidopsis thaliana*—seedlings	Polysome profiling	Sucrose feeding to Arabidopsis seedling induces selective mRNA translation events, which include numerous RP transcripts.
(Simm et al., 2015)	*Solanum lycopersicum*—Young leaves and anthers	Next generation sequencing, Quantitative real-time PCR (qRT-PCR)	Co-regulated clusters containing RBFs and RPs exert their functions preferentially in different tissues of *Solanum lycopersicum.*
(Moin et al., 2016)	*Oryza sativa*—Roots, shoots, leaves, root-shoot transition region, flowers, grains and panicles	Quantitative real-time PCR (qRT-PCR)	RP transcripts of the LSU are responsive to stress in *Oryza sativa*, suggesting that proteins encoded by these transcripts could play a specialized role responding to stress.
(Li et al., 2017a)	*Arabidopsis thaliana*	Labeled proteomics	Structural proteins of the LSU and SSU are stable and long-lived compared to other major protein complexes. Relative degradation rates of RPs had higher standard deviation, suggesting active remodeling takes place.
(Merret et al., 2017)	*Arabidopsis thaliana*—Seedlings	Polysome profiling, ^15^N elemental analysis mass spectrometry	Transcripts of uS12 (AtRPS23B), uS14 (AtRPS29B) and eL37 (AtRPL37B) are preferentially stored during heat shock and subsequently released and translated in an HSP101-dependent manner during recovery.
(Saha et al., 2017)	*Oryza sativa*—Plumules, radicles, shoot, and leaf	Quantitative real-time PCR (qRT-PCR)	RP transcripts of the SSU (RPS4, RPS13a, RPS18a and RPS4a) are upregulated during several abiotic stresses in *Oryza sativa*. RPS4 is also responsive to biotic stress.
(Belouah et al., 2019)	*Solanum lycopersicum—*Fruits	Transcriptome—proteome paired modelling	RP transcript translation (k_t_) and protein degradation rates (k_d_) are amongst the highest in all transcript-protein paired measurements of *Solanum lycopersicum* indicating flexible remodeling of cytosolic ribosomes.
(Sáez-Vásquez and Delseny, 2019)	Plants—review	Transcriptome data meta-analyses	Transcripts related to cytosolic ribosomes either of RAPs or of RPs are induced at transcriptome level by three major stresses, namely, cold, heat, and UV-B stress.
(Salih et al., 2019)	*Arabidopsis thaliana*	Labeled proteomics	Cytosolic ribosomal populations are replaced every 3-4 days according to the half-life of constituent RPs. RPs featuring significantly shorter turnover were P0D (RPP0D), 0.5 days and RACK1B and C, 1.2 days.
(Zhang et al., 2020)	*Arabidopsis thaliana*	Proteomics, Quantitative real-time PCR (qRT-PCR), reverse genetics	CKB1 functions in UV-B stress possibly by modulating the responses of the uL16 RP family paralogs of Arabidopsis.
(Cheong et al., 2020)	*Arabidopsis thaliana*	Transcriptome data, sucrose density ribosome purification, proteomics, reverse genetics	REIL proteins affect paralog composition of cytosolic ribosomes of Arabidopsis. The accumulation of non-translating and translating complexes, as well as their constituent RP transcript or proteoforms differ in REIL mutants.

Induced accumulation of RPs and RP paralogs exist in Arabidopsis. Label-free proteomics generated evidence of differential paralog use in response to changing physiological conditions. Phosphorous and iron deficiencies trigger differential accumulation of RPs in plant roots (Wang et al., 2013). UV-B treatment modulates the uL16 paralogs by increasing AtRPL10C and decreasing AtRPL10B (Ferreyra et al., 2010). This process is modulated by CKB1, i.e., the regulatory subunit of plastid Casein kinase2 (Zhang et al., 2020). Mutants of the cold-specific Arabidopsis RBF, REIL, indicated that ribosome biogenesis can alter RP paralog accumulation in non-translational ribosome complexes (Cheong et al., 2020). The abundance of specific paralogs, namely, eL28 (AtRPL28A) and eS7 (AtRPS7C), changed upon sucrose feeding (Hummel et al., 2012), and importantly, this effect of sucrose is concomitant to selective mRNA translation (Gamm et al., 2014). The causal link between both observations remains to be elucidated. When linking altered translation of RP paralogs to RP substoichiometry, claims of dosage compensation among plant paralogs within the respective RP family need to be carefully considered and dissected from potential paralog-specific functions. Examples indicate that subfunctionalization of RP paralogs exists. Arabidopsis paralogs of RP families uL16 (Falcone Ferreyra et al., 2013), uL23 (Degenhardt and Bonham-Smith, 2008a; Degenhardt and Bonham-Smith, 2008b), RACK1 (Guo and Chen, 2008) and uS8 (Szick-Miranda et al., 2010) are non-redundant in function, while other families, such as uL4, contain paralogs that can be linked to co-existing, potentially divergent populations of ribosomes (Rosado et al., 2010). Our compiled list of references implies the existence of translation bias mechanisms. Specialized ribosomes customized to environmental cues can contribute to such mechanisms (**Table 1**).

The influence of environmental and developmental cues on transcripts of plant cytosolic RP and RBF transcripts becomes increasingly evident. For Arabidopsis, a regulatory model that triggers ribosome heterogeneity was proposed based on transcriptome *in silico* analyses (Sormani et al., 2011b). Such a model assumes that plant ribosome heterogeneity plays a major role for the modulation and reprogramming of the translatome. In *Solanum lycopersicum* and *Brassica napus* clusters of RAPs determine tissue identity (Whittle and Krochko, 2009; Simm et al., 2015) and plant organ- or development-specific ribosomes are a well-known plant feature (Horiguchi et al., 2012). In *Oryza sativa* RP transcripts respond to abiotic stresses (Moin et al., 2016; Saha et al., 2017). The differential expression of RAP and specifically RP paralog genes implies that transcriptional reprogramming of the translatome mediates responses of the protein composition of ribosomes to environmental stimuli (**Figure 1** **Step 3**) but the contribution and interplay of transcription with additional layers of control of the protein composition of ribosomes require further research.

### Post-Translational Modifications

PTMs of RPs generate heterogeneous ribosomes without requiring *de novo* synthesis of complete ribosome complexes or synthesis of RPs followed by ribosome remodeling. In short, PTMs can create heterogeneity on a shorter time scale than possible by ribosome or RP turnover. Arabidopsis RPs undergo a great variety of covalent modifications, such as initiator methionine removal, N-terminal acetylation, N-terminal methylation, lysine N-methylation, phosphorylation and S-cyanylation (Carroll et al., 2008; Turkina et al., 2011; García et al., 2019). Non-targeted analysis of the CRP revealed more than one protein spot in a 2D gel proteomics analysis for half of the identified RPs and suggested the presence of multiple RP isoforms (Giavalisco et al., 2005). Presence of a variety of RP PTMs is further supported by proteomic studies where consideration of expected PTM mass shifts enhances peptide matching per RP family and even RP paralogs (Carroll et al., 2008).

In other eukaryotes, PTMs are involved in translational control (Simsek and Barna, 2017). The likely best investigated functional PTM of a plant RP is the TOR-mediated phosphorylation of the eS6 protein (AtRPS6). TOR is a eukaryotic master regulator complex that integrates energy and nutrient signaling at many system levels ranging from protein synthesis to the control of cell growth and proliferation (Xiong and Sheen, 2014; Chowdhury and Köhler, 2015). In plants, auxin is one of the main signals that affect TOR-mediated translational control (Schepetilnikov and Ryabova, 2017). Phosphorylation of the 40S ribosomal protein S6 kinase 1 (S6K1) is modulated by auxin upstream of TOR (Schepetilnikov et al., 2013) and in turn, leads to eS6 phosphorylation. Next to auxin, S6K1 is modulated by stimuli like glucose and light signals (Li et al., 2017b). The phosphorylation status of eS6 affects pre-18S rRNA synthesis at the rDNA level (Kim et al., 2014). Dephosphorylated eS6 directly binds to a plant-specific histone deacetylase that represses rDNA transcription by altering the chromatin structure. Additionally, translation reinitiation of specific ORFs relies on TOR/S6K1 activity (Schepetilnikov et al., 2011). In essence, eS6 phosphorylation and its upstream signaling cascade regulates translation at multiple levels by a direct link to a cellular master switch. Other plant examples include structural RPs that are differentially phosphorylated during the day and night cycle and modulate diurnal protein synthesis (Turkina et al., 2011), or P-stalk proteins that are phosphorylated and are thought to regulate translation initiation (Szick et al., 1998). Phosphorylation sites are known, e.g. Ser-103 of P1/P2 paralogs, RPP1A, 1B, and 1C, and Ser-305 of uL10 paralog, RPP0A (Reiland et al., 2009). Phosphorylation events at these sites may regulate selective translation in plants, as it appears to link an integrated stress response in mammalian models through the interaction with General control nonderepressible2 (GCN2) global translational regulator (Inglis et al., 2019).

In summary, diverse evidence of structural ribosome heterogeneity challenges the view of ribosomes as mere executing bystanders of protein synthesis. Observations of translational regulation by changes of translation initiation factors (eIFs) need to consider the multiple modes of ribosome heterogeneity. We think that there is reasonable doubt that ribosome heterogeneity is a mere consequence of stochastic ribosome assembly and that heterogeneity has the sole function of engineering redundancy to ensure a secure supply of the essential ribosome machinery. We support the view that evolution molded the ample structural diversity of plant ribosomes toward functionally specialized ribosomes, where PTM mechanisms act rapidly on slowly turned-over ribosome populations and *de novo* synthesis of ribosome complexes or RPs coupled to ribosome remodeling supports long-term acclimation to environmental changes.

## Functional Heterogeneity of RP Paralogs

For heterogeneous ribosomes to be functional, the translated proteome must be shaped by selective transcript recruiting according to external stimuli. Means of selective translation by structural changes to the ribosome at the RP level that became apparent in other organisms (Ferretti et al., 2017; Shi et al., 2017) remain to be proven in plants. One of the means that plants use to select subsets of transcripts for translation are cis-regulatory elements of mRNAs (Von Arnim et al., 2014; Van Der Horst et al., 2020). Ribosomes decode cis-regulatory elements. For instance, in Arabidopsis, RPL24B regulates uORF-mediated translation reinitiation at the 5´UTR (Nishimura et al., 2005). Through this mechanism, RPL24B modulates the auxin pathway during development, directing translation of auxin response factors (ARFs) (Rosado et al., 2012). Another example of how ribosomes rely on RPs to target subsets of mRNA is the RACK1 protein family. In yeast RACK1 affects translation in a length-dependent manner and promotes translation of short mRNAs (Thompson et al., 2016). The three Arabidopsis RACK1 paralogs proved to be functionally unequal (Guo and Chen, 2008). Although complementation studies and multi-paralog mutants indicate partial genetic redundancies, due to differential expression of the three paralogs, RACK1 factors have differential contributions to plant development and translation (**Table 2**). Thus, if selective translation is conserved, the paralogs might show distinct mRNA recruiting abilities. Remarkably, the knowledge gathered on this RP family by plant ribosome structural and functional research (Islas-Flores et al., 2015) contributed to the discovery of how poxviruses can achieve trans-kingdom mimicry by inducing a plant-like status of human RACK1 to translate their own RNA (Jha et al., 2017). These examples show that plant RP paralogs can functionally diversify. In the following, we surveyed further evidence of functional heterogeneity of plant RPs that may reach beyond cytosolic ribosomes (**Table 2**).

**Table 2:** Studies of structural ribosomal protein mutant lines of *Arabidopsis thaliana*, *Oryza sativa*, *Nicotiana tabacum*, and *Nicotiana benthamiana* sorted by RP family.

Gene code	Paralog	Family	Phenotype
**Cytosolic ribosome**			
**Os03g0725000**	RPL6—OsRPL6	eL6	Two mutants with high water-use efficiency in rice (Moin et al., 2017).
**AT4G27090**	RPL14B	eL14	Heterozygous female gametophytes from *rpl14b*/RPL14B ovules are impaired for cell fate specification resulting in pollen tube defects (Luo et al., 2020).
**AT5G27850**	RPL18C	eL18	Pointed leaves (Horiguchi et al., 2011) and reduced leaf area (Wang et al., 2018).
**AT1G02780**	RPL19A	eL19	Embryo lethal (Tzafrir et al., 2004).
**AT3G16780**	RPL19B—NbRPL19	eL19	Decreased non-host disease resistance against bacterial pathogens (Nagaraj et al., 2016).
**AT2G34480**	RPL18aB	eL20	Required for both male gametophyte function and embryo development (Yan et al., 2016).
**AT2G36620**	RPL24A	eL24	Suppresses proline accumulation of the parental *Arabidopsis thaliana* ring zinc finger 1 (atrzf1) mutant (Park et al., 2017).
**AT3G53020**	RPL24B	eL24	Pale leaf-color (Yao et al., 2008), defects in gynoecium apical–basal patterning, RP paralogs with different translational status (Nishimura et al., 2005; Tiruneh et al., 2013).
**AT2G19730**	RPL28A	eL28	Serrated-pointed leaves (Horiguchi et al., 2011), pale leaf color (Yao et al., 2008).
**AT3G59540**	RPL38B	eL38	Larger palisade mesophyll cells coupled with serrated-pointed leaves (Horiguchi et al., 2011).
**AT4G31985**	RPL39C	eL39	Pointed leaves (Horiguchi et al., 2011).
**AT3G52590**	RPL40B	eL40	Embryo lethal (Tzafrir et al., 2004).
**AT3G23390**	RPL36aA	eL42	Serrated-pointed leaves (Casanova-Sáez et al., 2014).
**AT4G14320**	RPL36aB	eL42	Serrated-pointed leaves (Casanova-Sáez et al., 2014).
**AT2G27530**	RPL10aB	uL1	Serrated-pointed leaves (Pinon et al., 2008) (Horiguchi et al., 2011).
**AT2G18020**	RPL8A	uL2	Embryo lethal (Tzafrir et al., 2004).
**AT1G43170**	RPL3A	uL3	Embryo lethal (Tzafrir et al., 2004), silencing uL3 genes in *Nicotiana tabacum* affects growth (Popescu and Tumer, 2004).
**Os11g0168200**	RPL3B	uL3	Paralog A does not compensate mutation in paralog B, reduction in free 60S subunits and polysomes, aberrant leaf morphology (Zheng et al., 2016), silencing uL3 genes in *Nicotiana tabacum* affects growth (Popescu and Tumer, 2004).
**AT3G09630**	RPL4A	uL4	Aberrant auxin responses and developmental phenotypes (Rosado et al., 2010; Rosado et al., 2012).
**AT5G02870**	RPL4D	uL4	Abaxialized leaves with larger palisade mesophyll cells (Horiguchi et al., 2011), defects in vacuole trafficking and development, downregulation of genes implicated in lipid metabolism (Li et al., 2015), uORF-mediated translation repression of SAC51 by sac52-d (AtRPL10A), sac53-d (AtRACK1A), sac56-d (AtRPL4D), and thermospermine (Kakehi et al., 2015).
**AT1G33140**	RPL9C	uL6	Serrated-pointed leaves with laminar outgrowths (Pinon et al., 2008), delayed growth, paralogs C and D have redundant functions (Devis et al., 2015).
**AT4G10450**	RPL9D	uL6	Delayed growth, paralogs C and D have redundant functions (Devis et al., 2015).
**AT5G60670**	RPL12C—NbRPL12	uL11	Decreased non-host disease resistance against bacterial pathogens (Nagaraj et al., 2016).
**Os01g0348700**	RPL23A—OsRPL23A	uL14	Two mutants with high water-use efficiency in rice (Moin et al., 2017).
**AT3G04400**	RPL23C	uL14	Embryo lethal (Tzafrir et al., 2004).
**AT2G47110**	RPL27aB	uL15	Female gametogenesis less strongly affected than in aC paralog mutant (Zsögön et al., 2014).
**AT1G70600**	RPL27aC	uL15	Serrated-pointed leaves, embryo and plant shoot developmental defects (Szakonyi and Byrne, 2011), female sterility (Zsögön et al., 2014).
**AT1G14320**	RPL10A	uL16	Female gametophyte lethality (Imai et al., 2008), embryo lethal (Ferreyra et al., 2010), uORF-mediated translation repression of SAC51 by sac52-d (AtRPL10A), sac53-d (AtRACK1A), sac56-d (AtRPL4D) and thermospermine (Kakehi et al., 2015).
**At1G26910**	RPL10B	uL16	knock down mutant, reduced growth in all measured physiological parameters (Ferreyra et al., 2010).
**AT3G25520**	RPL5A	uL18	Reduced female/male transmission (Fujikura et al., 2009), serrated-pointed leaves and reduced leaf development (Wang et al., 2018). Abnormal, similar to abaxialized leaves when combined with *as1* (Pinon et al., 2008) or *as2*, otherwise wild-type like (Yao et al., 2008).
**AT5G39740**	RPL5B	uL18	Abnormal, similar to abaxialized leaves when combined with *as2*, otherwise pale coloring (Yao et al., 2008). Reduced female/male transmission (Fujikura et al., 2009), functionally redundant to RPL5A paralog and decreased leaf width (Van Minnebruggen et al., 2010).
**AT2G39460**	RPL23aA	uL23	RNAi line, pointed and fused leaves, delayed flowering, retarded plant growth, apical dominance loss, lethal double-mutant with paralog aB (Degenhardt and Bonham-Smith, 2008a), low levels of RPL23A amiRNA result in an albino phenotype (Kim et al., 2015).
**AT3G55280**	RPL23aB	uL23	No phenotype reported, lethal double-mutant with paralog aA (Degenhardt and Bonham-Smith, 2008a), low levels of RPL23A amiRNA result in an albino phenotype (Kim et al., 2015).
**AT1G80750**	RPL7A	uL30	Pointed leaves and reduced leaf area (Wang et al., 2018).
**AT2G01250**	RPL7B	uL30	Serrated-pointed leaves (Horiguchi et al., 2011) and reduced leaf area and development (Wang et al., 2018).
**AT4G31700**	RPS6A	eS6	Strongest phenotype within eS6 family, has been combined with *as1* and *as2* mutants (Horiguchi et al., 2011), reduced leaf area and enhanced *var2*-mediated leaf variegation (Wang et al., 2018), slow growth (haplodeficiency) in paralog A-B double mutant (Creff et al., 2010).
**AT5G10360**	RPS6B	eS6	Defective phyllotaxy, apical dominance loss (Morimoto et al., 2002), slow growth (haplodeficiency) in paralog A-B double mutant (Creff et al., 2010).
**AT5G41520**	RPS10B	eS10	Affects the formation and separation of shoot lateral organs, including the shoot axillary meristems (Stirnberg et al., 2012)
**AT3G53890**	RPS21B	eS21	Serrated-pointed leaves, reduced leaf area (Wang et al., 2018) and cell size in shoot (Horiguchi et al., 2011).
**AT5G27700**	RPS21C	eS21	Functionally redundant to B paralog, reduced leaf area (Wang et al., 2018).
**AT5G28060**	RPS24B	eS24	Serrated-pointed leaves, reduced leaf area (Wang et al., 2018).
**AT3G61110**	RPS27A	eS27	Increased sensitivity to UV-B and methyl methanesulfonate (Revenkova et al., 1999).
**AT5G03850**	RPS28B	eS28	Decreased cell proliferation, has been combined with *as1* and *as2* mutations (Horiguchi et al., 2011).
**AT3G11940**	RPS5A	uS7	Cell-division perturbed when heterozygous, embryo lethal when homozygous (Weijers et al., 2001).
**AT2G19720**	RPS15aB	uS8	Pointed leaves, a double mutant with RPL28A was investigated (Horiguchi et al., 2011), type II uS8, evolutionarily divergent and plant specific paralog (Szick-Miranda et al., 2010).
**AT4G29430**	RPS15aE	uS8	Type II uS8, evolutionarily divergent and plant specific paralog, larger leaf surface, root, and cells (Szick-Miranda et al., 2010).
**AT1G22780**	RPS18A	uS13	Pointed leaves and reduced growth (Van Lijsebettens et al., 1994).
**AT4G00100**	RPS13A	uS15	Defects of leaf and trichome morphology, retarded flowering and root growth (Ito et al., 2000).
**AT3G48930**	RPS11A	uS17	Embryo lethal (Tzafrir et al., 2004).
**AT1G18080**	RACK1A	RACK1	Pointed leaf phenotype and partial genetic redundancy of paralogs by complementation studies (Guo and Chen, 2008), AtRPL4D restored by sac52-d (AtRPL10A), sac53-d (AtRACK1A), sac56-d and thermospermine (Kakehi et al., 2015).
**AT1G48630**	RACK1B	RACK1	No phenotype reported, exacerbates RACK1A mutation defects (Guo and Chen, 2008).
**AT3G18130**	RACK1C	RACK1	No phenotype reported, exacerbates RACK1A mutation defects (Guo and Chen, 2008).
**Plastid ribosome**			
**Os01g0662300**	RPL12	bL12c	Albino lethal phenotype at seedling stage (Zhao et al., 2016).
**Os02g0259600**	RPL21/CL21	bL21c	Chloroplast developmental defects and seedling death in rice, the synonymous mutant name is *asl2* (*albino seedling lethality 2*) (Lin et al., 2015).
**Os01g0749200**	RPL13A	uL13c	Single-base substitution affects chloroplast development in rice grown under low temperature conditions (Song et al., 2014), albino lethal-seedlings of T-DNA insertion mutant (Lee et al., 2019).
**AT3G25920**	RPL15	uL15c	Decreased levels of uL15c lead to chlorosis and reduced leaf photosynthetic capacity, the null mutant is embryo lethal (Bobik et al., 2019).
**AT5G54600**	RPL24	uL24c	Reductions in growth, leaf pigments and photosynthesis (Romani et al., 2012).
**AT5G30510**	RPS1	bS1c	Reductions in growth, leaf pigments and photosynthesis (Romani et al., 2012).
**Os12g0563200**	RPS6	bS6c	Albino phenotype at low temperature (Wang et al., 2018), pale leaves and defective thylakoid architecture (Sun et al., 2016).
**Os01g0678600**	RPS20	bS20c	Albino lethal phenotype at seedling stage (Gong et al., 2013).
**AT2G38140**	PSRP-4	bTHXc	Putative role in light-dependent regulation of translation (Tiller et al., 2012).
**AT3G52150**	PSRP-2	cS22	Putative role in light-dependent regulation of translation (Tiller et al., 2012).
**AT1G68590**	PSRP-3	cS23	Putative role in light-dependent regulation of translation (Tiller et al., 2012).
**AT2G33800**	RPS5	uS5c	Smaller rosettes, photosystem I and II components, and many PRPs are suppressed, involved in plant development and cold stress (Zhang et al., 2016).
**Os03g0769100**	RPS9	uS9c	Embryo lethal in maize (Ma and Dooner, 2004), albino at three leaf stage in rice (Qiu et al., 2018).
**AT1G79850**	RPS17	uS17c	Reductions in growth, leaf pigments and photosynthesis (Romani et al., 2012), embryo-lethal in maize (Schultes et al., 2000).

To reduce ambiguity of interpretation, we avoided to report mutants of multiple defective loci where unique RP mutant lines were available; information of combined mutants is indicated.

### Cytosolic Ribosomal Proteins

Many RP genes have been mutated to enable a deeper functional understanding of their gene products. These studies focus on the developmental role of single or few RP paralogs (Horiguchi et al., 2011; Horiguchi et al., 2012). A summary of studies that target the functions of single RPs tell a story of common themes and diversity (**Table 2**). Diversity becomes apparent, for example, by observations that distinct developmental stages need specific RP paralogs, e.g., the uL23 (AtRPL23) paralogs, which are not equivalent for plant development (Degenhardt and Bonham-Smith, 2008a). In addition, the loss of single RP paralogs often causes phenotypes of varying severity, questioning claims of full functional RP paralog redundancy.

On the other hand, common phenotypes are apparent (**Table 2**). The *rp* mutants share typical features, for example, altered leaf polarity establishment, cell proliferation and shape determination (Byrne, 2009; Horiguchi et al., 2011; Roy and Arnim, 2013) and the frequent occurrence of embryo-lethality. The latter observation is in agreement with the essential function of ribosomes. Early embryo development is achieved through the use of inherited ribosomes, but the embryo cannot advance further because ribosome *de novo* synthesis is necessary. Where the mutation is not embryo lethal, a pointed leaf phenotype is frequently found. The shared *rp* phenotypes can be explained by ribosome insufficiency, i.e., the limited availability of translationally competent ribosomes, or alternatively by the lack of developmentally specialized ribosome subpopulations. In the plant field, however, the extra ribosomal, or so-called moonlighting functions of RPs, such as detailed for human pathogenesis mechanisms (Wang et al., 2015), are frequently considered explanations for *rp* mutant phenotypes (Gerst, 2018). These non-structural functions of RPs are just beginning to be unveiled and may be independent of ribosome specialization (Segev and Gerst, 2018). Systematic functional analyses of *rp* paralog mutants need to account for such extra-ribosomal functions of RPs. For instance, uL23 recruits a nascent protein to its future localization in the chloroplast by coupling with its receptor (Kim et al., 2015). Whether the differential uL23 paralog phenotypes are influenced by both moonlighting and ribosomal functions remains an open discussion.

In summary, the unambiguous experimental dissection of the three basic functional explanations of RP deficiencies, namely, ribosome specialization, ribosome insufficiency, or moonlighting of single RPs with functions that are linked to ribosome biogenesis or translation, is the grand challenge of the field of plant ribosome physiology.

### Plastid Ribosomal Proteins

The cyanobacterial origin of chloroplasts determines the nature of their 70S bacterial-type ribosomes. Part of the chloroplast proteome comprising ~3000 proteins, is nuclear encoded (Jensen and Leister, 2014), while only 100 ORFs remain chloroplast encoded (Jarvis and López-Juez, 2013). Consequently, final protein abundances in the chloroplast are mostly determined posttranscriptionally, translationally, and posttranslationally (Sun and Zerges, 2015). Recent years have observed increasing interest in the plastid translational apparatus. The first structure of the spinach chloroplast ribosome was made available in 2016 using cryo-EM (Bieri et al., 2017). Insights into ribosome-associated factors were rapidly facilitated by the structure modeling capabilities of cryo-EM technology (Ahmed et al., 2017; Bieri et al., 2017; Graf et al., 2017; Boerema et al., 2018).

Plastid-specific ribosomal proteins (PSRPs) are split between nuclear and plastid encoded and can be divided in the model plant Arabidopsis into essential and nonessential components (Tiller et al., 2012). By definition, the nonessential components of chloroplast ribosomes are a subset of proteins that can be removed without an obvious phenotype. These nonessential accessory proteins may represent specialized factors that are needed beyond optimized *in vitro* or controlled greenhouse conditions. The severity of the *psrp* mutant phenotypes does not strictly correlate with their orthologous prokaryotic counterparts (Romani et al., 2012). This observation suggests plant-specific features of plastid ribosome biogenesis and translation. The functional studies reported in **Table 2** are selections of snapshots that highlight plant-specific aspects of plastid ribosomes. PSRP families are typically smaller than cytosolic RP families, and some PSRPs appear to be single copies. Future research will determine whether concepts of ribosome heterogeneity, specialization, and insufficiency or PSRP moonlighting may apply to plastid ribosomes that acclimate to environmental stress, such as cold stress. Plants appear to modify plastid ribosomes at suboptimal temperatures. In Arabidopsis, tolerance to cold can be achieved by overexpression of plastid ribosomal proteins, e.g., uS5c (PRSP5) (Zhang et al., 2016). PRPS5 and PRPS1 interact indirectly with the CHLOROPLAST RIBOSOME ASSOCIATED (CRASS) protein to support translation during cold stress (Pulido et al., 2018). In rice, uL13c is important for plastid development during cold (Song et al., 2014).

### Mitochondrial Ribosomal Proteins

The number of plant mito-*rp* studies is small as compared to those analyzing genes of the cytosolic or plastidic ribosomes. Nevertheless, the already existing body of literature, reviewed elsewhere (Robles and Quesada, 2017), points toward mito-RP families with functionally divergent members across plant species. Moreover, mito-RPs have particular roles during development (Robles and Quesada, 2017) that still need to be linked to either moonlighting functions or to their translational context. Interestingly, single-particle cryo-EM images in combination with proteomic analyses of enriched Arabidopsis mitochondrial ribosome fractions have shown substantial structural divergence from their prokaryote and eukaryote counterparts (Rugen et al., 2019; Waltz et al., 2019). The current body of studies suggests plant-specific features of mitochondrial translation.

## Transcriptomic Evidence of Plant Ribosome Specialization: A Test-Case

This section exemplifies and critically assesses instances of differential paralog usage that can be observed at the transcript level. Final protein abundance is shaped at several control points ranging from chromatin modifications to transcription, translation and PTMs (Vogel and Marcotte, 2012). Hence, transcript levels cannot predict the final active protein concentration or nature and extent of PTMs. Nevertheless, transcript changes are arguably a crucial component of the translational response to environmental cues in plants (see *Deviation From Canonical Compositions* and **Table 1**). In yeast, a mechanism that remains to be probed in plants relies on regulating the transcription of RPs in response to arrested ribosome biogenesis (Albert et al., 2019). Hence, we argue that changes in RP transcript levels provide one line of evidence—in the sense of a translation potential (**Figure 1** Step 3) or feedback mechanisms—that supports the search for ribosome specialization in the context of stress acclimation.

**Figure 3:**
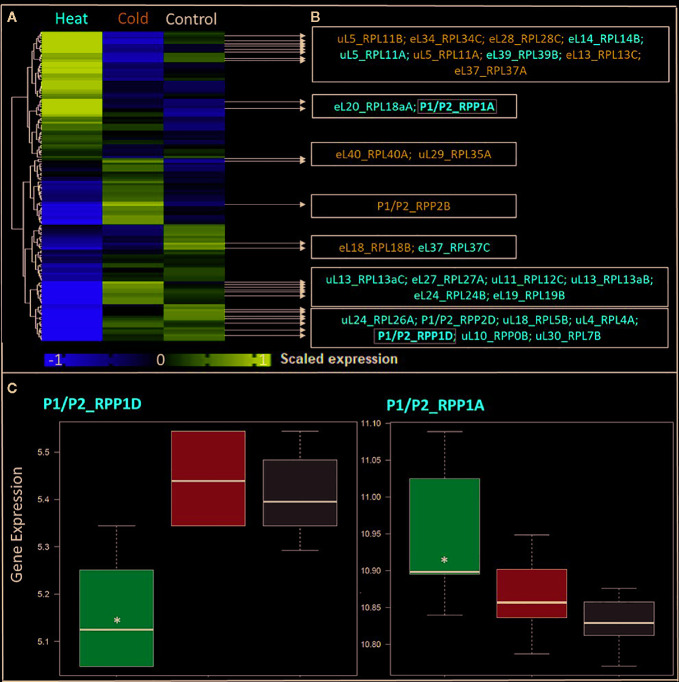
Differential expression in response to temperature stress of Arabidopsis genes encoding structural ribosomal proteins of the 60S subunit. Expression values of 96 40S and 136 60S RPs and RP paralogs were retrieved from Genevestigator experiment AT-00120 of root tissue (n = 30) exposed to 38°C heat shock (n = 6) or 4°C cold stress (n = 12) compared to 20°C control conditions [n= 12]. Transcriptome data processing and analysis was carried out in the R programming language and environment for statistical computing, https://www.R-project.org/ (Ihaka and Gentleman, 1996), using the R project for statistical computing (RRID : SCR_001905). Gene intensities were background subtracted and quantile normalized. **(A)** Heatmap of autoscaled and treatment-scaled abundances ranging from -1 (yellow) to +1 (purple), mean centering was performed by the function colMeans with the R package timeSeries (version 3042.102; https://CRAN.R-project.org/package=timeSeries). Correlation and Euclidean distance produced equivalent heatmaps due to the mode of scaling. R packages ComplexHeatmap (Gu et al., 2016), circlize (Gu et al., 2014) and fBasics (version 3042.89; https://CRAN.R-project.org/package=fBasics) were used to transform data and create the Heatmap. The statistical significance of changed gene expression relative to the control was evaluated by a generalized linear model (GLM). **(B)** False discovery rate (FDR)-corrected, significant temperature-responsive transcripts following heat shock (red, 20 genes) or cold stress (blue, 10 genes). One example of inversely regulated expression of two members of a single RP family, i.e., P-stalk components, during heat is highlighted in bold and by a gray outline (* in panel **C**). **(C)** Boxplots of the highlighted inversely heat responsive transcript abundances of the P1/P2 paralogs RPP1D and RPP1A. Data are log_2_-transformed, background subtracted, quantile normalized, and non-scaled.

To substantiate this claim, we chose temperature stress acclimation as a test case and show that differential gene expression may indicate changes of ribosome paralog composition as one mechanism of generating functional heterogeneity adjusted to environmental cues (**Figure 3**). Exploring temperature stress was the obvious choice in view of the increasingly visible effects of global warming. We propose a meta-analysis of the dynamics of RP family transcripts following opposing temperature shifts. In our case study, we compare *Arabidopsis thaliana* Col-0 root exposed to heat shock (38°C) and cold (4°C) stress with the respective control at 20°C optimized temperature. The experiment is identified as entry AT-00120 in the Genevestigator repository (organism: *Arabidopsis thaliana*, selection: AT-8, type: Gene). We based our test-case on a compiled list of 376 Arabidopsis genes that have been annotated as members of the cytosolic ribosomal proteome. As to the procedures, the background-subtracted microarray signals of experiment AT-00120 were retrieved and imported into the R statistical programming environment. All initial matrix related transformations, object conversions and data handling were performed with the R packages stringi (version 1.4.6—https://cran.r-project.org/web/packages/stringi/index.html), reshape (Wickham, 2007), and Tidyverse (version 1.0.0—https://github.com/hadley/tidyverse). Only the signals belonging to heat or cold stress subset of AT-00120 were further processed, because suboptimal temperature was reported to impact significantly RBF and RP transcripts (Sáez-Vásquez and Delseny, 2019). The resulting matrix was quantile normalized using the R package preprocessCore (version 1.46.0 - https://github.com/bmbolstad/preprocessCore). Afterwards the distribution of the data within treatments and genes was evaluated with density plots for treatments using the R package ggplot2 (Ginestet, 2011), and a Cullen and Frey graph for ATGs using the R package fitdistrplus (Delignette-Muller and Dutang, 2015). Analysis of the distribution patterns determined that a generalized linear model (GLM) was the appropriate statistical test. Accordingly, GLMs were fitted with different link functions to parametrize the mean and variances. Gamma, Lognormal and Gaussian functions were applied. The ranking and significances of resulting *P* values did not differ among link functions, showing the robustness of quantile normalization of data. Significance values were corrected for multiple testing using the false discovery rate (FDR) approach (Benjamini and Hochberg, 1995) and a significance threshold of *P* < 0.05 applied to all analyses.

**Figure 4:**
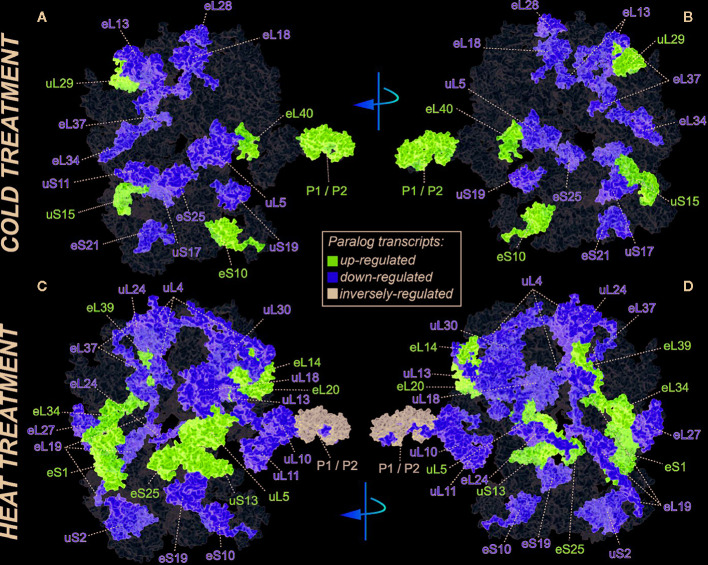
RP remodeling potential of Arabidopsis 80S ribosomes upon 4°C cold stress **(A**, **B)** or 38°C heat stress **(C**, **D)**. The visualization outlines mapped changed transcript abundances in response to temperature stress compared to optimized control conditions, i.e., ~ 20°C. Transcript data were statistically evaluated across individual paralogs within RP families as reported in **Figure 3** and mapped to a 3D rendering of the wheat 80S monosome (Armache, 2011) using the rotated 2D positions of **Figure 2**. Homology of wheat and Arabidopsis RP families was confirmed by protein BLAST matching. Black color indicates RP families with inversely regulated paralog transcripts following either cold or heat stress. Yellow and purple represent RP families with increased or decreased transcript abundances of at least one of the RP paralogs. The paralog identities and temperature specific transcript changes are reported in **Supplemental Table S2**.

We selected a test case of RP gene expression in root tissue because this tissue is often neglected in temperature studies, even though root systems of crops are frequently exposed to temperature extremes (Kaspar and Bland, 1992). Normalized gene expression intensities were divided in 60S and 40S subunit coding genes according to a curated list of the Arabidopsis cytosolic ribosomal proteome (**Supplemental Table S1**). Abundances were auto-scaled in order to plot them in a heatmap with equal means and variances (Gu et al., 2014; Gu et al., 2016) (**Figure 3A**). The Arabidopsis RP names were used as identifiers in the heatmap to highlight paralog-specific behavior of transcripts. Each ribosomal protein family was scored to belong to one of three response groups. Group (1) was defined as “increased”, if transcripts of one or more paralogs within a RP family were significantly increased. Group (2) was defined as “decreased”, if transcripts of one or more paralogs within a RP family were significantly decreased. Finally, group (3) was defined as “inversely regulated”, if a transcript of at least one paralog was significantly increased and in parallel another paralog of the same RP family was significantly decreased, either under heat or under cold stress (**Figures 3A–C**). To visualize the spatial distribution and location within the 80S ribosome, the increased, decreased, and inversely regulated RP families were mapped onto the previously outlined 3D representation of the 80S wheat monosome (**Figure 2**), applying different color codes to the significantly changed RP families (**Figure 4**). For the mapping PyMOL visualization software (RRID : SCR_000305) was used to obtain a surface representation and to highlight proteins with significant changes. By choice of 2D rotations, emphasis was given to the proteins that are visible from either the interface- or solvent-sides. In the interest of simplifying the image, rRNAs were excluded from the structural representation. The expression patterns were reduced from paralogs in **Figure 3** to RP-family level in **Figure 4** for the sake of visualization and reflect the RP paralog specific behaviors reported in **Supplemental Table S2**.

This meta-analysis adds the new aspect of differential RP paralog usage to the plethora of insights gathered on plant system reprogramming during temperature acclimation at metabolic, transcript or protein levels, e.g. (Scharf and Nover, 1982; Merret et al., 2017; Calixto et al., 2018; Beine-Golovchuk et al., 2018). Considering the significant observations only from this exemplary study, heat shock may induce more changes than cold acclimation (**Figure 3**). The results indicate fundamental temperature-specific reprogramming of RP gene transcription (**Figures 3A, B** **)** and differential responses among cytosolic RP families under opposing conditions of temperature stress. Focusing on the changes that occur within RP families, we encountered instances of potential temperature-specialized RP paralogs, and, as exemplified by the inversely regulated P1/P2 P-stalk components AtRPP1D and AtRPP1A, even indications of paralog preference under heat stress (**Figures 3C** and **4C, D**).

Some of the significant gene expression changes from our test case have been reported and investigated previously. For example, the transcript encoding for eL37 (AtRPL37B) is sequestered into stress granules upon heat stress to be quickly released during stress deacclimation to resume cytosolic ribosome synthesis (Merret et al., 2017). This process limits availability of eL37 (AtRPL37B) transcripts for translation under heat stress. Sequestration into stress granules stores and recycles transcripts and therefore does not require changes of total mRNA. Our meta-analysis indicates an additional regulation of the eL37 family at total mRNA level. Significant transcript changes of two different eL37 paralogs, AtRPL37a and AtRPL37C, occur following heat stress and are also part of cold acclimation (**Figures 3** and **4**).

RP transcript levels of Arabidopsis roots are, however, not necessarily associated with compositional changes of the non-translating ribo-proteome (Cheong et al., 2020). Ribosomes are stable complexes and have a longer half-life as compared to average protein half-lives. Ribosomes may share this property with other multi-protein complexes as plant enzymes embedded in complexes have a significantly longer half-life than free enzymes (Nelson et al., 2013). Mammalian RPs have a longer half-life in the cytoplasm, where RPs can be considered to be enriched in the ribosome-bound state, as compared to RPs of the nucleolus, where higher fractions of free RPs are expected to support the assembly process (Boisvert et al., 2012). A ribosome half-life range of ~ 72-178 h is reported for normal and regenerating rat liver (Hirsch and Hiatt, 1966; Nikolov et al., 1983). A similar range of 3-4 days was determined for Arabidopsis ribosomes (Salih et al., 2019). Therefore, it can be argued that transcript changes will need to extend over long periods to alter overall RP or RP paralog abundances or, alternatively, transcript changes will only affect the subpopulation of newly synthesized ribosomes. Cytosolic ribosomes may remodel surface accessible RPs upon environmental cues, e.g., by paralog loss and addition or by exchange processes. Such variation may explain why cytosolic plant RPs have significantly higher standard deviations of mean degradation rates as compared to other large plant protein complexes (Li et al., 2017a). In contrast to the long ribosome half-life, the variance of total RP degradation rates does not differ compared to the average variance of proteins that are not part of large complexes. Higher turnover of individual cytosolic RPs may favor the quick appearance of new ribosomal populations by restructuring the cellular CRP population rather than by a complete degradation and *de novo* synthesis cycle. Therefore, we suggest that altered transcript abundances may rapidly translate into altered CRP stoichiometry. Studies aiming to characterize translatome-reprogramming at the onset of stress acclimation should reveal differential dynamics of RP and RP paralog synthesis and incorporation into the translating CRP.

## Future Perspectives

Evidence of ribosome specialization by differential paralog use alone (e.g., Yu et al., 2019) or in combination with the other modes of structural variation is currently considered a hard problem in science (Haag and Dinman, 2019). Functional specialization of ribosomes, ribosome insufficiency, and the moonlighting functions of RPs (e.g., Kyritsis et al., 2019) in their non-ribosome-bound state are difficult to differentiate and likely are not mutually exclusive. For example, 1) an RP or RP paralog that gives rise to a substoichiometric ribosome subpopulation may have a moonlighting function in its free state or 2) the absence of a ribosome subpopulation lacking a specialized RP paralog or combination of paralogs from different RP families, may cause partial insufficiency. Clearly, future studies of RP paralogs must consider and characterize ribosome heterogeneity and test potential constraints of translation for effects of ribosome insufficiency and for control by moonlighting functions (Ferretti and Karbstein, 2019).

Despite the complexity of investigating ribosome heterogeneity and specialization, new technologies make tackling this hard problem feasible (Emmott et al., 2019). These technologies fill the gap of knowledge between the transcriptome and the acting proteome. Ribosome profiling methods support claims of ribosome specialization by monitoring actively translated mRNAs. This variant of transcript analysis sequences the ~30 nucleotide footprints that are protected by 80S translating ribosomes (Ingolia et al., 2009; Juntawong et al., 2015; Hsu et al., 2016; Ingolia et al., 2019). Ribosome profiling or footprinting is an improved proxy of transcript translation compared to total mRNA profiling. This technique answers questions related to transcripts that are ribosome-bound under given experimental conditions, revealing the distribution of monosomes along translated transcripts and allowing to spot translational stalling events. Furthermore, improvements relative to RNA-seq partly rely on excluding mRNA that is part of inactive transcript pools. Examples of excluded transcripts include those sequestered in stress granules for transient storage or associated with processing bodies and subject to catabolism (Chantarachot and Bailey-Serres, 2018; Lee and Seydoux, 2019), as well as the contribution of the nuclear transcriptome and incompletely spliced pre-mature mRNAs (Lee and Bailey-Serres, 2018). Ultimately, transcript complexity is reduced by more than 50% when polysomal-bound mRNAs are sequenced (Zhang et al., 2015). In essence, Ribosome profiling, aka Ribo-Seq, provides improved insight into the translated transcriptome (Wang and Sachs, 1997; Seidelt et al., 2009; Ishimura et al., 2014; Hou et al., 2016; Matsuo et al., 2017; Yamashita et al., 2017; Zhang et al., 2017).

Additional information that is required to link the actively translated transcriptome to the proteome may be obtained by measuring *de novo* protein synthesis through label-assisted proteomics. Label-assisted proteomic studies monitor the kinetics of label incorporation into proteins, allowing for calculations of protein synthesis and degradation rates at biological steady states (Nelson and Millar, 2015). Under optimized conditions, label incorporation can be used as direct evidence of *de novo* ribosome biosynthesis or remodeling by condition-specific RP paralogs. In the first case, the tracer incorporation into total ribosome complexes can be measured after amino acid hydrolysis and isotope enrichment analysis. These measurements can be used to calculate rates of *de novo* ribosome biosynthesis. In the latter case, calculations of individual RP paralog turnover (Salih et al., 2019) indicate which RPs and RP paralogs are *de novo* synthesized and which paralogs are recycled from pre-existing ribosomes.

Moving beyond biological steady states will be required to reveal whether environmental cues trigger ribosome heterogeneity. To do so, current turnover studies using a metabolic tracer to label RP paralogs have to be refined. The limitations that need to be overcome include intrinsic properties of plant cytosolic ribosomes. First, the unusually high variance of degradation RP rates (Li et al., 2017a) suggests that the stoichiometry of the ribosome complexes or paralog composition may change. Consequently, controls are necessary that verify or detect remodeling and deviations of ribosome complexes from the canonical structure (**Figures 2** and **4**). Second, paralog-resolved ^15^N-dependent turnover analysis of RPs is possible (Salih et al., 2019; Salih et al., 2020), but the dynamics of label incorporation into soluble amino acids pools need to be taken into account. Environmental stresses including temperature stress affect metabolites and change pool sizes of free amino acids, e.g., (Kaplan et al., 2004). The rate of label incorporation into amino acid monomers will change in response to environmental cues. If the free amino acid pools are not carefully considered, observed differential label incorporation rates into RPs or RP paralogs may be misinterpreted. Third, non-translating and translating fractions of ribosome complexes exist that may harbor different quantities of pre-existing and *de novo* synthesized ribosome. Separation of the diverse pools of ribosome complexes, e.g. (Beine-Golovchuk et al., 2018), will enhance our insight and answer questions on complex specific or non-specific label incorporation by calculating protein turnover of plant RPs in the non-translating compared to translating ribosomal fractions.

Finally and as a general consideration, functional heterogeneity research in plants will enhance sustainability in agriculture. Rice and maize are plant models and crops in which ribosome biology is already well understood. In rice, the paralog OsL23A, homolog of AtRPL23A, was shown to positively affect the drought and salt stress responses (Moin et al., 2017). Moreover, ribosome heterogeneity is apparently triggered by environmental stress in rice (Moin et al., 2016; Moin et al., 2017). This observation indicates that ribosome heterogeneity may be generalized. Crops engineered at ribosome level may be of utmost importance for future food security. In maize, the tool box of ribosome profiling has been refined (Chotewutmontri et al., 2018), and insights into translatome regulation during drought stress (Lei et al., 2015) and viral infection (Xu et al., 2019) have been gained. RP abundance and phosphorylation status change during germination in maize (Hernández-Hermenegildo et al., 2018) and provide the potential of selective mRNA translation by heterogeneous ribosome populations during seedling development. Similarly, specific clusters of tomato RBFs and RPs are differentially expressed and are characteristics of tissue identity (Simm et al., 2015). These studies indicate the importance of future functional RP studies for diminishing the effects of climate stress on crop production.

## Summary

Given the highly variable nature of the plant ribosomal proteome and the availability of many experimental tools in the plant field (Merchante et al., 2017), plants may have extraordinary potential for the study of structural and functional ribosome heterogeneity at RP level. The high number of plant paralogs per RP family compared to other eukaryote models warrants explanation and in depth analysis of potential paralog specialization that can contribute to a plant ribosomal code. Current research indicates that plant ribosome populations are heterogeneous at multiple levels. Deviations of the canonical ribosome structure by substoichiometry, additional interacting proteins, PTMs of rRNA and RPs, or rRNA variants, and by multiple RP paralogs are known. The functional consequence of ribosome heterogeneity, however, is in many cases a matter of debate, but ribosome specialization has been proven in some cases, for instance in the case of the TOR-mediated control of ribosome function by secondary modification of the RPS6 structural protein (Kim et al., 2014; Chowdhury and Köhler, 2015; Dobrenel et al., 2016). A wealth of functional analyses of plant RPs indicates that the structural variation potential of the multiple RPs or paralogs may have functions in plant development and physiology. The involvement of RP paralogs in plant stress physiology is supported by current literature (**Table 2**). Differential gene expression analysis of high versus low temperature responses, where RP gene expression is largely inverted and the balance between paralogs of RP families can be changed (**Figures 3** and **4**) supports the notion of plant ribosome specialization.

Ribosomes must translate specific subsets of mRNA species to be considered functionally specialized. The intrinsic potential of ribosome remodeling and *de novo* synthesis to produce ribosome populations adapted to control translation of mRNA subsets needs to be further investigated. Such research has been applied to developmental biology as well as to stress physiology across many model organisms (Bailey-Serres, 1999; Kawaguchi et al., 2004; Branco-Price et al., 2005; Nicolaï et al., 2006; Branco-Price et al., 2008; Mustroph et al., 2009; Matsuura et al., 2010; Sormani et al., 2011a; Juntawong and Bailey-Serres, 2012; Moeller et al., 2012; Ueda et al., 2012; Wu et al., 2012; Xue and Barna, 2012; Browning and Bailey-Serres, 2015; Thompson et al., 2016; Jha et al., 2017; Shi et al., 2017; Simsek et al., 2017; Bates et al., 2018; Genuth and Barna, 2018; Guo, 2018; Emmott et al., 2019; Mageeney and Ware, 2019; Sulima and Dinman, 2019; Yu et al., 2019). However, questions of functional conservation, convergence, or speciation across organism kingdoms remain largely unanswered. Full explanation of a ribosomal code will likely reveal synergies of mechanisms and may require concomitant exchanges of RPs, involvement of ribosome associated factors, changes of rRNA status, PTMs, and ribosome biogenesis or ribosome remodeling. All in all, we hypothesize that all ribosome functions, i.e., ribosome biogenesis, translation initiation, elongation, termination, and recycling, may be affected by ribosome heterogeneity.

## Data Availability Statement

Publicly available datasets were analyzed in this study. This data can be found here: Genevestigator, AT-00120.

## Author Contributions

FM-S: Conceptualization, literature research, figure shaping, statistical analyses, structural analysis and mapping, manuscript writing. OB-G: Ribosomal proteome supplement, literature research, figure shaping. Y-CH: Figure shaping, structural analysis and mapping. JK: Conceptualization, literature research, figure shaping, manuscript writing.

## Funding

We acknowledge the Max-Planck Society (Max Planck Institute of Molecular Plant Physiology) and the University of Melbourne for funding this research via the Melbourne-Potsdam PhD Programme (MelPoPP).

## Conflict of Interest

The authors declare that the research was conducted in the absence of any commercial or financial relationships that could be construed as a potential conflict of interest.

## Abbreviations

RP, Ribosomal Protein; ORF, Open Reading Frame; PTM, Posttranslational Modifications; GLM, Generalized Linear Model; FDR, False Discovery Rate; LSU, Large Subunit; SSU, Small Subunit; RAP, Ribosome Associated Proteins; RBF, Ribosome Biogenesis Factors; CRP, Cytosolic Ribosomal Proteome; PSRP, Plastid-Specific Ribosomal Proteins; Cryo-EM, cryogenic electron microscopy.
